# „Lipid rescue“ als Bail-out-Strategie bei einem mehrfach reanimierten Patienten

**DOI:** 10.1007/s00063-025-01292-7

**Published:** 2025-06-18

**Authors:** Jana Ackmann, Judit Grans-Siebel, Christoph Hüser, Volker Burst, Christoph Adler

**Affiliations:** 1 Medizinische Fakultät und Uniklinik Köln, Klinik III für Innere Medizin, Universität zu Köln, Kerpener Str. 62, 50937 Köln, Deutschland; 2 Medizinische Fakultät und Uniklinik Köln, Klinik I für Innere Medizin, Universität zu Köln, Kerpener Str. 62, 50937 Köln, Deutschland; 3 Medizinische Fakultät und Uniklinik Köln, Klinik II für Innere Medizin, Universität zu Köln, Kerpener Str. 62, 50937 Köln, Deutschland; 4 Klinik für Akut- und Notfallmedizin, Klinikum Leverkusen, Am Gesundheitspark 11, 51375 Leverkusen, Deutschland

## Anamnese

Ein 32-jähriger männlicher Patient wurde in Notarztbegleitung mit dem Verdacht auf eine Mischintoxikation in unserer Notaufnahme vorgestellt. Er wurde zuvor vigilanzgemindert auf dem Boden eines Nachtclubs aufgefunden. Andere Gäste beobachteten, wie er Alkohol, Lachgas (N_2_O) und weitere Drogen, mutmaßlich Kokain, konsumiert hatte, sodass unter anderem von einer Kokainintoxikation ausgegangen wurde. Über die medizinische Vorgeschichte lagen keine Informationen vor. Bei Eintreffen des Rettungsdiensts im Nachtclub war der Patient bewusstlos (Glasgow Coma Score, GCS: 3). Im Folgenden kam es unmittelbar nach Eintreffen des Rettungsdiensts 2‑malig zu einem hypodynamischen Herz-Kreislauf-Stillstand (HKS) bei Asystolie. Nach wenigen Zyklen kardiopulmonaler Reanimation (CPR) konnte jeweils eine Rückkehr des Spontankreislaufs („return of spontaneous circulation“, ROSC) erreicht werden. Der Patient wurde komplikationslos intubiert, maschinell beatmet und mit stabilen Kreislaufverhältnissen in die Uniklinik Köln transportiert. Noch vor dem Umlagern im Schockraum kam es erneut zu einem HKS bei Asystolie. Bei unklarer Identität des Patienten lagen keine weiteren medizinischen Informationen vor.

## Untersuchung

In der klinischen Untersuchung bei Übernahme zeigte sich folgender Befund:

- A: endotracheal intubiert (Tubus 8 mm), Lunge beidseits belüftet;
- B: maschinell beatmet (Volume Controlled Ventilation, VCV): Fraction of Inspired Oxygen (F_i_O_2_) 100 %, Atemfrequenz (AF) 15/min, Positive End-Expiratory Pressure (PEEP): 8 mmHg, SpO_2_ 98 %;
- C: HKS bei Asystolie, laufende Reanimation;
- D: GCS 3, Pupillen: weit und lichtstarr;
- E: Temperatur 37,4 °C, keine Verletzungen oder Einstichstellen.

## Diagnostik

Die initiale venöse Blutgasanalyse (BGA) unter Reanimation zeigte eine schwere Hyperkaliämie (7,9 mmol/l) und eine kombinierte respiratorische und metabolische Acidose (pH 6,915) mit einem pCO_2_ von 98 mm Hg, einem Base Excess (BE) von −12,3 mmol/l und einem Laktat von 15,66 mmol/l.

## Wie lautet Ihre Diagnose?

## Therapie und Verlauf

**Abb. 1:**
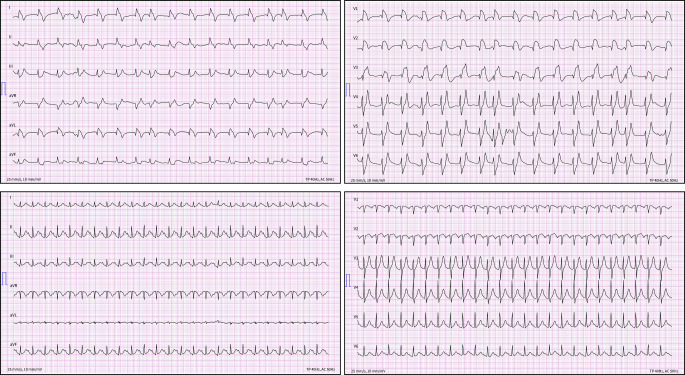
12-Kanal-EKG vor (*oben*) und nach Lipidinfusion (*unten*)

**Abb. 2:**
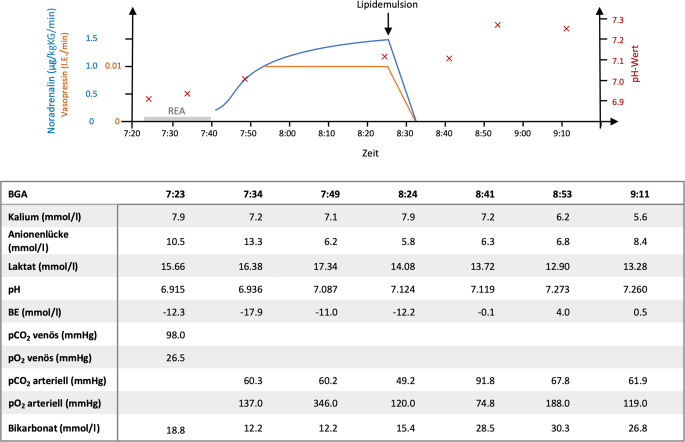
Katecholamine, pH-Wert und ausgewählte Werte der Blutgasanalysen

Wir pufferten die Acidose wiederholt mit 8,4 % Natriumhydrogencarbonat, verabreichten Glukose/Insulin, Kalziumgluconat und Salbutamol per Inhalation. Außerdem erhielt der Patient probatorisch Naloxon und Flumazenil, was jedoch zunächst keine Wirkung zeigte. Im Verlauf der Reanimation entwickelte der Patient Kammerflimmern. Nach 3‑maliger Defibrillation und kumulativ 18-minütiger Reanimation nach ERC-Standard wurde eine ROSC erreicht. Das EKG zeigte Vorhofflimmern und diffuse ST-Strecken-Hebungen (Abb. 1). Die linksventrikuläre Pumpfunktion (LV-Funktion) war global in mehreren Kontrollen leichtgradig eingeschränkt, Rechtsherzbelastungszeichen zeigen sich nicht, ein Perikarderguss konnte ausgeschlossen werden. Das FAST (Focused Assessment with Sonography for Trauma) zeigte keine freie Flüssigkeit. Trotz der Maßnahmen blieb der Patient hämodynamisch instabil mit rapide steigendem Katecholaminbedarf (Noradrenalin bis 1,5 µg/kgKG/min und Vasopressin 0,01 IE/min) nach Reanimation, sodass wir bereits Vorbereitungen zur extrakorporalen kardiopulmonalen Reanimation (eCPR) mittels extrakorporaler Membranoxygenierung (ECMO) trafen. Aufgrund der weiter steigenden Katecholamine unter supportiver Therapie und fehlender kausaler therapeutischer Standardoptionen entschieden wir uns bei V. a. Intoxikation mit Kokain für die Infusion einer Lipidemulsion (SMOFlipid® 20 %, Fresenius Kabi, Bad Homburg, Deutschland; initialer Bolus von 100 ml [ca. 1,5 ml/kgKG], gefolgt von 150 ml als Infusion). Hierunter kam es zur unmittelbaren hämodynamischen Stabilisierung des Patienten, der innerhalb von 6 min nach Bolusgabe katecholaminfrei war (Abb. 2). Auf eine ECMO konnte dementsprechend verzichtet werden. Das EKG zeigte zu diesem Zeitpunkt einen Sinusrhythmus, der QRS-Komplex war schmal und die ST-Strecken-Veränderungen regredient.

In der erweiterten Diagnostik zeigte sich eine kraniale Computertomographie (cCT) unauffällig, eine Computertomographie (CT) der Lunge zeigte eine Aspirationspneumonie ohne anderen wegweisenden Befund. Im Verlauf zeigte sich laborchemisch im Urin wider Erwarten kein Nachweis von Kokain, sondern der Suchtest (Immunoassay; möLab, Langenfeld, Deutschland) war positiv für Methamphetamin, Amphetamin und Methylendioxy-N-methylamphetamin (MDMA). Weiterhin bestand eine manifeste Hyperthyreose (TSH: < 0,01 mU/l, fT3: 18,1 ng/l, fT4: 35,5 ng/l) bei erhöhten TSH-Rezeptor-Antikörpern (TRAK: > 6 IU/l, Normwert < 1,75 IU/l). Der Blutalkoholspiegel lag bei 0,18 ‰. Der Patient wurde auf die Intensivstation verlegt, erhielt eine thyreostatische Therapie und eine Dialyse. Die linksventrikuläre Pumpfunktion war bereits im kurzfristigen Verlauf hyperkontraktil. Im weiteren Verlauf entwickelte der Patient eine schwere Rhabdomyolyse mit einer initialen Kreatinkinase (CK) von 1694 U/l und einer maximalen CK von 117.040 U/l am Folgetag sowie eine akute Nierenfunktionsstörung, sodass die Dialyse fortgesetzt werden musste. Nach einigen Tagen zeigte sich in der neurologischen Diagnostik eine schwere hypoxische Hirnschädigung, sodass die Therapie aufgrund der infausten neurologischen Prognose in Rücksprache mit den Angehörigen im Sinne des mutmaßlichen Patientenwillens schließlich eingestellt wurde.

## Diskussion

Wir stellen einen Fall einer lebensbedrohlichen Intoxikation bei einem hämodynamisch instabilen, mehrfach reanimierten Patienten vor, der sich nach intravenöser Gabe einer Lipidemulsion innerhalb kürzester Zeit vollständig stabilisierte. Der angenommene Wirkmechanismus der Lipidemulsion ist ein „lipid sink“ mit der Bildung einer Plasmalipidphase, in die sich lipophile Wirkstoffe bevorzugt bewegen, und ein folgender Shuttle-Mechanismus für die Substanzen zur Leber, der ihre Metabolisierung und Eliminierung fördert [1]. Es gibt auch Hinweise darauf, dass die Lipidemulsion intrazelluläre Mechanismen beeinflusst, darunter die Verbesserung der mitochondrialen Dysfunktion, die Modulation von Signalkaskaden und eine potenzielle Umkehr einer kardialen Natriumkanalblockade [1].

Initial gingen wir bei ausstehenden Laborergebnissen und unscharfen fremdanamnestischen Angaben von einer Kokainintoxikation aus. Lipide gelten als Therapieoption bei versehentlicher intravenöser Injektion von Lokalanästhetika, die sich vom Kokain ableiten [1], und es gibt Fallberichte über den Einsatz einer Lipidemulsion bei schwerer Kokainintoxikation [2–4]. Auch der bei unserem Patienten positive Suchtest für Methamphetamin, Amphetamin und MDMA weist prinzipiell lipophile Substanzen nach, die für ihre Wirksamkeit die Blut-Hirn-Schranke überwinden müssen. Limitierend gibt es hierbei eine hohe Kreuzreaktivität des Tests und es wurde kein Bestätigungstest durchgeführt, sodass die genaue Substanz unklar bleibt. Für eine Amphetamin- und Metamphetaminintoxikationen sind beim Hund positive Effekte einer Lipidemulsion beschrieben worden [5, 6]. Für den Menschen gibt es unserer Kenntnis nach bisher einen Fallbericht über die erfolgreiche Behandlung einer Methamphetaminintoxikation bei einem 55-Jährigen Patienten [7]. In diesem Fallbericht konnte die Stabilisierung der Kreislaufverhältnisse 20 min nach Bolusgabe erreicht werden.

Beim dargestellten Fall zeigten die Laborparameter zum Aufnahmezeitpunkt eine ausgeprägte metabolische Acidose mit begleitender Hyperkaliämie. Vor dem Hintergrund der massiv ansteigenden CK-Werte ist es plausibel, dass eine beginnende Rhabdomyolyse im Rahmen der Intoxikation zur Elektrolytentgleisung beigetragen hat. Diese könnte wiederum das initiale Reanimationsereignis, insbesondere die beobachtete pulslose elektrische Aktivität (PEA) und die hypodynamischen Kreislaufverhältnisse, mitverursacht oder aggraviert haben. Ein solches Bild ist für Stimulanzienintoxikationen eher untypisch, bei gleichzeitiger Hyperkaliämie jedoch erklärbar. Im weiteren Verlauf kam es zu Kammerflimmern, das möglicherweise durch die Stimulanzien selbst oder möglicherweise auch durch die Gabe von Antidoten, wie Flumazenil und Naloxon, begünstigt worden sein könnte.

Hinzu kommt in unserem Fall, dass der Patient eine thyreotoxische Krise im Rahmen einer Erstdiagnose eines Morbus Basedow hatte. Ein Fallbericht beschreibt eine Patientin mit methamphetamininduzierter Hyperthyreose [8], sodass auch in dem hier berichteten Fall eine substanzvermittelte Exazerbation der Hyperthyreose möglich scheint. Welchen Einfluss eine hyperthyreote Stoffwechsellage letztendlich hatte, bleibt unklar.

Die rapide hämodynamische Stabilisierung nach Lipidgabe spricht in unserem Fall zwar für deren Effektivität, andere denkbare Ursachen für die Stabilisierung müssen jedoch auch bedacht werden.

Die Gabe von Adrenalin im Rahmen der Reanimation wirkt kaliumsenkend und könnte die intermittierende ROSC erklären. Ein weiterer denkbarer Stabilisierungsmechanismus, neben der Senkung des Kaliums, könnte der parallele (partielle) Ausgleich der metabolischen Acidose durch Natriumbikarbonat gewesen sein. Allerdings blieb der pH-Wert in der Stabilisierungsphase aufgrund der respiratorischen Komponente der Acidose weiterhin niedrig, sodass hier ein multifaktorieller Mechanismus wahrscheinlicher ist. Ein akutes Koronarsyndrom oder ein kardiogener Schock können prinzipiell auch mit Stimulanzienmissbrauch assoziiert sein. Echokardiographisch zeigten sich allerdings keine Wandbewegungsstörungen, auch ergaben sich im Verlauf keine Hinweise auf eine Ischämie oder Kardiomyopathie. Die linksventrikuläre Pumpfunktion war initial leichtgradig reduziert, wobei eine vorübergehende myokardiale Dysfunktion nach prolongierter Reanimation nicht unüblich ist [9], besserte sich jedoch schnell und war bereits wenige Stunden nach dem initialen Ereignis hyperkontraktil. Auch zeigten sich nach Elektrolytausgleich keine ischämietypischen EKG-Veränderungen. Die initiale Asystolie könnte prinzipiell auch durch eine Hypoxie ausgelöst worden sein, insbesondere im Hinblick auf die beim Eintreffen des Rettungsdiensts unbekannte Dauer der bereits vorliegenden Bewusstlosigkeit. Die späteren Reanimationsereignisse und hämodynamische Instabilität wären jedoch bei stabilen pO_2_-Werten nicht durch eine Asphyxie allein erklärbar.

Insgesamt war die schnelle Kreislaufstabilisierung innerhalb weniger Minuten nach der Bolusgabe der Lipidemulsion auffällig, was sich pharmakologisch durch den Lipid-sink-Mechanismus erklären lässt. Da andere begleitende therapeutische Maßnahmen bereits zuvor eingeleitet wurden, legt der enge zeitliche Zusammenhang einen wesentlichen therapeutischen Effekt der Lipidgabe nahe, auch wenn die supportive Therapie insbesondere der Ausgleich von Acidose und Hyperkaliämie sicherlich ebenfalls einen Beitrag zur Stabilisierung leisteten.

**Diagnose:** Herz-Kreislauf-Stillstand infolge Stimulanzienintoxikation mit ausgeprägter metabolischer Azidose und Hyperkaliämie

Ein zu bedenkender Aspekt im Kontext der Lipidemulsion ist eine mögliche Einschränkung technischer Notfallmaßnahmen. Bei Patienten mit Bedarf für eine va-ECMO kann eine Lipidämie die Funktion des Systems beeinträchtigen und beispielsweise zu Funktionsstörung des Oxygenators führen, was den Einsatz der va-ECMO verzögern oder einschränken könnte [10]. In unserem Fall war eine va-ECMO nicht erforderlich, dieser Punkt sollte jedoch bei zukünftigen Fällen beachtet werden.

## Fazit für die Praxis

- Bisher wurden nur einige wenige Fallberichte über den Einsatz von Lipidemulsionen bei Intoxikationen mit psychoaktiven Substanzen am Menschen veröffentlicht.
- Bei Patienten mit einer solchen Intoxikation und rhythmischer oder hämodynamischer Instabilität, die auf Standardmaßnahmen nicht ansprechen, könnten intravenöse Lipide als Bail-out Strategie jedoch von Nutzen sein.
